# Hepatoprotective Effects of Insect Extracts in an Animal Model of Nonalcoholic Fatty Liver Disease

**DOI:** 10.3390/nu10060735

**Published:** 2018-06-07

**Authors:** A-Rang Im, Won-Kyung Yang, Yang-Chun Park, Seung Hyung Kim, Sungwook Chae

**Affiliations:** 1 Herbal Medicine Research Division, Korea Institute of Oriental Medicine, 1672 Yuseong-daero, Yuseong-gu, Daejeon 34053, Korea; lar747@kiom.re.kr; 2 Institute of Traditional Medicine and Bioscience, 62 Daehak-ro, Dong-gu, Daejeon University, Daejeon 34520, Korea; ywks1220@dju.kr (W.-K.Y.); sksh518@dju.ac.kr (S.H.K.); 3 Division of Respiratory Systems, Department of Internal Medicine, College of Korean Medicine, Daejeon University, 176 Daeduk-ro, Seo-gu, Daejeon 35235, Korea; omdpys@dju.kr; 4 Korean Medicine Life Science, University of Science and Technology, 217 Gajeong-ro, Yuseong-gu, Daejeon 34113, Korea

**Keywords:** nonalcoholic fatty liver disease, edible insect, *Protaetia brevitarsis seulensis*, *Oxya chinensis sinuosa*, *Gryllus bimaculatus*, hepatoprotection

## Abstract

Insects represent the largest and most diverse group of organisms on earth and are potential food and drug resources. Recently, we have demonstrated that a *Forsythia viridissima* extract prevented free fatty acid-induced lipid accumulation in an in vitro cellular nonalcoholic fatty liver disease (NAFLD) model. In this study, we aimed to evaluate the hepatoprotective effects of extracts of the insects *Protaetia brevitarsis seulensis* Kolbe, 1886 (PB), *Oxya chinensis sinuosa* Mishchenko, 1951 (OC), and *Gryllus bimaculatus* De Geer, 1773 (GB) in a high-fat diet (HFD)-induced NAFLD animal model, as well as to elucidate the underlying mechanisms. The effects of the supplementation with PB, OC, and GB extracts were evaluated histopathologically and histochemically. PB, OC, and GB extract supplementation inhibited the HFD-induced increase in body weight and body fat mass and ameliorated other adverse changes, resulting in decreased liver function parameters, lower serum triglyceride and cholesterol levels, and increased serum adiponectin levels. The expression of hepatic genes involved in lipid droplet accumulation and in fatty acid uptake also decreased upon treatment of HFD-fed mice with the extracts. These results provide evidence of the protective effects of the PB, OC, and GB extracts against HFD-induced fatty liver disease in an animal model.

## 1. Introduction

Nonalcoholic fatty liver disease (NAFLD) is a common chronic liver disease, which encompasses the entire spectrum of fatty liver diseases occurring in individuals in the absence of significant alcohol consumption and ranging from fatty liver to steatohepatitis and cirrhosis. NAFLD is generally considered a comorbidity of obesity and a hepatic manifestation of metabolic syndrome [1,2]. Currently, NAFLD has reached epidemic proportions and represents the most common cause of chronic liver disease in the community [3].

NAFLD is generally considered to result from an increased delivery and uptake of free fatty acids (FFA) into hepatocytes, owing to their excessive dietary intake or release from the adipose tissue, increased de novo hepatic FFA and triglyceride (TG) synthesis, failure of very low-density lipoprotein (LDL) synthesis and TG export, or failure of FFA elimination due to impaired hepatic mitochondrial β-oxidation [4]. Under physiological conditions, TG synthesis is stimulated to dispose of excess FFAs, leading to excessive accumulation of hepatic TGs, which is associated with an increased supply of FFAs from the peripheral adipose tissue to the liver and to an enhanced de novo lipid synthesis via the lipogenic pathway [5]. Thus, the circulating pool of FFAs increases in obese individuals and accounts for most of liver TGs in NAFLD [6].

Insects represent the largest and most diverse group of organisms on earth, making up 80–90% of the world’s biodiversity. They have been considered potential food and drug resources, and this traditional knowledge may contribute to the future development of insects as food ingredients worldwide [7]. Because of the increasing global population and decreasing availability of arable land, it is essential to select and develop additional food and feed resources, and insects are an important potential source of food and feed [8].

*Protaetia brevitarsis seulensis* (Kolbe, 1886), used as a traditional Korean medicine, has been temporarily registered as a food material by the Ministry of Food and Drug Safety of Korea for treating diverse diseases, such as breast cancer, inflammatory disease, hepatic cancer, liver cirrhosis, and hepatitis [9,10,11]. *Oxya chinensis sinuosa* (Mishchenko, 1951), a grasshopper species belonging to the phylum Arthropoda, has long been used as food in Asia. Although there is little information on its chemical constituents or their activities, some studies have shown its antithrombotic and antiplatelet effects [12,13]. *Gryllus bimaculatus* (De Geer, 1773) has also been demonstrated to show anti-inflammatory effects, and a study has suggested that a glycosaminoglycan obtained from *G. bimaculatus* may be useful for the treatment of inflammatory diseases, including chronic arthritis [14]. 

The aim of our study was to evaluate the hepatoprotective effects of ethanol extracts of three insects, including *P. brevitarsis seulensis* (PB), *O. chinensis sinuosa* (OC), and *G. bimaculatus* (GB), in a high-fat diet (HFD)-induced animal NAFLD model, as well as to elucidate the underlying mechanisms.

## 2. Materials and Methods

### 2.1. Preparation of PB, OC, and GB Extracts

Dried PB, OC, and GB were purchased from a commercial supplier. All voucher specimens were deposited at the herbal bank of the Korea Institute of Oriental Medicine. Dried PB, OC, and GB were pulverized and extracted with 70% ethanol. The crude extract solutions were filtered, evaporated, and lyophilized in a freeze dryer.

### 2.2. Cell Culture and Treatment

HepG2 cells were obtained from the American Type Culture Collection (Manassas, VA, USA) and were maintained in a 1:1 mixture of Dulbecco’s modified Eagle’s medium and F-12 medium (Invitrogen, Carlsbad, CA, USA), supplemented with 1% penicillin/streptomycin (Gibco, Grand Island, NY, USA) and 10% heat-inactivated fetal bovine serum (Invitrogen), at 37 °C in an atmosphere containing 5% CO_2_. PB, OC, and GB extracts cytotoxicity was assessed using the 3-(4,5-dmethylthiazol-2-yl)-5-(3-carboxymethoxyphenyl)-2-(4-sulfophenyl)-2H-tetrazolium inner salt (MTS) assay. HepG2 cells in 96-well plates were treated with varying PB, OC, and GB extracts concentrations. After incubation for 24 h, the MTS solution (CellTiter Aqueous One Solution, Promega, Madison, USA) was added to each well, and the plates were incubated at 37 °C for 4 h. The optical absorbance was determined at 490 nm with a microplate spectrophotometer (Molecular Devices, Sunnyvale, CA, USA).

Oleic acid (OA) and palmitic acid (PA) were purchased from Sigma-Aldrich (St. Louis, MO, USA) and were prepared in 0.1 M NaOH at 70 °C, followed by filter sterilization. A 1 mM FFA mixture (0.66 mM OA and 0.33 mM PA) was prepared with bovine serum albumin (BSA) at a final concentration of 1% in the culture medium. The cells were starved with Dulbecco’s modified Eagle’s medium and F-12 (50:50; Invitrogen, Carlsbad, CA, USA) supplemented with 1% penicillin/streptomycin (P/S, Gibco, Grand Island, NY, USA) and 0.5% heat-inactivated fetal bovine serum (Invitrogen) at 37 °C in an atmosphere containing 5% CO_2_ for 24 h. After starvation, the cells were treated with 1 mM FFA for 24 h. The cells were used when they reached 75% confluence.

### 2.3. Nile Red and Oil Red O Staining

Nile red, a fluorescent hydrophobic dye, was applied to demonstrate the presence of phospholipids in HepG2 cells. The intracellular fat content was determined using Oil Red O (Sigma, St. Louis, MO, USA), a lipophilic dye used to detect fat accumulation in the cytosol. After 24 h of FFA exposure, with or without the PB, OC, and GB extracts, the cells were washed twice with phosphate-buffered saline (PBS) and then incubated with 0.75 μg/mL Nile red dye for 15 min at room temperature. To minimize dye photobleaching, the plates were wrapped in aluminum foil. The fluorescence intensity was measured using a SpectraMax M2 fluorescence spectrophotometer (Molecular Devices) at an excitation wavelength of 485 nm and an emission wavelength of 572 nm. For Oil Red O staining, the cells were exposed to FFA for 24 h, with or without the PB, OC, and GB extracts, washed twice with PBS, and fixed with 10% formalin for 1 h. The cells were stained with an Oil Red O solution and then examined under a light microscope. After observing the presence of lipid droplets, 100% isopropanol was added to each well, and the fluorescence was measured at 520 nm using a spectrophotometer (Molecular Devices). Each treatment was performed in triplicate.

### 2.4. Measurement of Intracellular TG Content

The cellular TG content was measured enzymatically with a commercial Fuji Dri-Chem 3500 kit (Fujifilm, Tokyo, Japan) following the manufacturer’s instructions. The TG content was expressed in micrograms of TGs per microgram of protein. Each treatment was performed in triplicate. 

### 2.5. Animals and Treatment

Male C57BL/6J mice were purchased from Dae Han Bio Link Co. (Eumsung, Korea). All mice were kept under controlled temperature (22 ± 1 °C) and humidity (55 ± 10%) on a 12 h light–dark cycle. For NAFLD studies, the mice were randomly divided into nine groups of 13 animals each, with an equal distribution of the body weight: a control group, a HFD group, a HFD plus 100 mg/kg/day milk thistle (MT) extract group, a HFD plus 100 mg/kg/day PB group, a HFD plus 200 mg/kg/day PB group, a HFD plus 100 mg/kg/day OC group, a HFD plus 200 mg/kg/day OC group, a HFD plus 100 mg/kg/day GB group, and a HFD plus 200 mg/kg/day GB group. The mice in the normal group were fed a Purina diet with 10% of calories derived from fat (38057, Koatech, Seoul, Korea). The mice in the HFD groups were fed a pellet rodent diet with 60% of total calories derived from fat (D12492, Research Diets Inc., New Brunswick, NJ, USA). The animals were sacrificed for analysis after 14 weeks. The mice were fasted overnight prior to being sacrificed, with blood samples collected from the anesthetized mice via cardiac puncture. The serum was separated from the blood cells by centrifugation and was stored at −80 °C until analysis. The liver and other tissues were excised, rinsed with PBS to remove the blood, and snap-frozen in liquid nitrogen. The tissue samples were also stored at −80 °C until analysis. All animal procedures were performed in accordance with the guidelines for the Care and Use of Laboratory Animals developed by the Institute of Laboratory Animal Resources of the National Research Council and were approved by the Institutional Animal Care and Use Committee of Daejeon University (Daejeon, Korea). 

### 2.6. Histopathological Examinations and Oil Red O Staining

For histopathological examination, the abdomen was opened, and the liver was quickly removed to obtain fresh liver tissue specimens, which were fixed in 10% formalin and prepared as paraffin blocks. Sections from the blocks were stained with hematoxylin and eosin (H&E). To determine the hepatic lipid droplet accumulation, frozen sections of liver tissue were stained with Oil Red O. The sections were visualized under a CKX41 microscope (Olympus, Tokyo, Japan), and digital images were captured at a magnification of 200× using the Image Plus 2.0 program (Motic, Causeway Bay, Hong Kong, China). The adipose tissues were separated into abdominal subcutaneous fat, epididymal adipose tissue, kidney adipose tissue, and visceral fat, and the weight of each tissue specimen was measured. 

### 2.7. Triglyceride Accumulation Assay

The liver triglyceride (TG) content was measured enzymatically using a commercial kit (Asan Pharmaceutical Co., Seoul, Korea) according to the manufacturer’s instructions. The TG content in the liver tissues was expressed as micrograms of TG per milligram of liver tissue. 

### 2.8. Serum Chemistry Analysis

Samples of the cardiac whole blood collected from anesthetized animals after an overnight fast were centrifuged at 2000× *g* for 15 min at 4 °C to separate the serum from the blood cells. The serum biochemical concentrations of TGs, total cholesterol (TC), LDL cholesterol, high-density lipoprotein (HDL) cholesterol, glucose, and creatinine were determined using an automatic analyzer (Hitachi-720; Hitachi Medical, Tokyo, Japan) with reagents purchased from Bio-Clinical System (Gyeonggi-do, Anyang, Korea). The serum concentrations of FFAs, alanine aminotransferase (ALT), and aspartate aminotransferase (AST) were measured using an XL-200 automatic clinical chemistry analyzer (Erba Diagnostics, Mannheim, Germany). Serum adiponectin and insulin-like growth factor 1 (IGF-1) levels were measured by an enzyme-linked immunosorbent assay (ELISA) using commercially available kits (Linco Research, Inc., St. Charles, MO, USA). The blood serum was placed in the wells of a microplate coated with a diluted antibody and was incubated at 4 °C overnight. Each well was washed three times with a buffer, followed by the addition of 100 μL of plasma (10-fold dilution), incubation for 1 h at room temperature, and two subsequent washes with a buffer. Then, 100 μL of a streptavidin–horseradish peroxidase conjugate was added, and the plate was incubated for 1 h at room temperature, followed by a wash. Subsequently, 100 μL of a tetramethylbenzidine substrate was added, and the plate was incubated in the dark for 30 min, followed by the addition of 50 μL of a stop solution. The absorbance was measured at 450 nm using an ELISA reader. 

### 2.9. RNA Isolation and Quantitative Real-Time Polymerase Chain Reaction

RNA was isolated from 30 mg of liver tissue samples using TRIzol (Invitrogen) according to the manufacturer’s instruction. Reverse transcription and quantitative real-time polymerase chain reaction (qPCR) were performed to determine the relative mRNA expression using TaqMan probes (Applied Biosystems, Foster City, CA, USA).

### 2.10. Protein Extraction and Western Blotting

The lysates of liver tissue prepared from 100 µg of liver tissue after fat removal, and aliquots of the supernatant containing 20 µg of proteins were subjected to 10% SDS-PAGE. After electrophoresis, the proteins were blotted onto polyvinylidene fluoride (PVDF) membranes. The membranes were incubated overnight at 4 °C with specific antibodies for the proteins of interest and for β-actin. The immunoblots were visualized using an ECL chemiluminescence detection system (Bio-Rad, Hercules, CA, USA), and the bands were visualized using a chemiluminescence imaging system (ImageQuant LAS 4000 mini, GE Healthcare, Buckinghamshire, UK). The primary antibodies used were anti-SREBP1c, -Fsa27/Cidec (Abcam, Cambridge, UK), -leptin, -adiponectin, -pACC, -Fabp4/AP2, -UCP2, -PPARγ, -β-actin (Cell Signaling Technology, Danvers, MA, USA), followed by secondary antibodies (Cell Signaling Technology, Danvers, MA, USA).

### 2.11. Determination of TNF-α and IL1β Secretion by ELISA

TNF-α and IL1β (R&D Systems, Minneapolis, MN, USA) levels in the adipose tissue were determined using ELISA kits, according to the manufacturer’s instructions. The levels of TNF-α and IL1β were quantified by colorimetric analysis. 

### 2.12. Statistical Analysis

All experimental data are shown as mean values ± standard error of the mean (SEM). Between-group differences were evaluated using one-way analysis of variance, followed by Tukey’s test, to detect significant differences between the HFD and the control groups. 

## 3. Results

### 3.1. Effects of Insect Extracts on FFA-Induced Lipid Accumulation in HepG2 Cells

**Figure 1:**
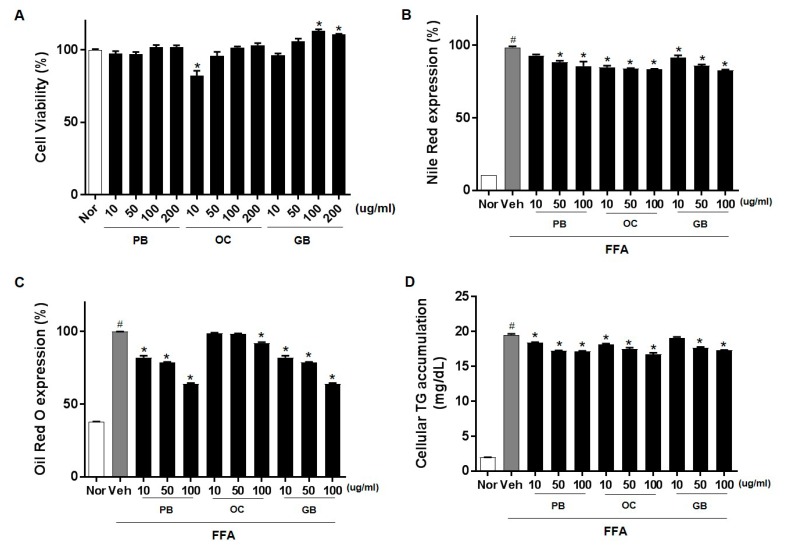
Inhibition of free fatty acids (FFA)-induced lipid accumulation by *Protaetia brevitarsis seulensis* (PB), *Oxya chinensis sinuosa* (OC), and *Gryllus bimaculatus* (GB) extracts in HepG2 cells. HepG2 cells were cultured with 0.1% bovine serum albumin (BSA, control), 1 mM FFA mixture, or 1 mM FFA and PB, OC, and GB extracts (10, 50, and 100 μg/mL) for 24 h. (**A**) Cell viability after 24 h of treatment with PB, OC, and GB extracts. After 24 h, intracellular lipid accumulation was assessed by Nile red staining (**B**) and Oil Red O staining (**C**). The data are representative of three independent experiments. (**D**) Intracellular triglyceride (TG) content was measured, and absorbance readings were normalized to protein concentrations. Nor, normal. The data represent the mean ± SEM of at least three individual experiments; # *p* < 0.05 compared with the control group; * *p* < 0.05 versus FFA alone.

Hepatic steatosis develops because of the dysregulation of one or multiple lipid-associated pathways. To investigate the efficacy of the PB, OC, and GB extracts against hepatic steatosis, we used an FFA-induced in vitro cellular hepatic steatosis model. We induced lipid accumulation in HepG2 cells by supplementing the cells with common plasma FFAs, namely, PA and OA, which mimic the excessive FFA influx into hepatocytes during NAFLD. After treatment with PB, OC, and GB extracts, the samples displayed no apparent cytotoxic effect at concentrations up to 200 μg/mL (Figure 1A). We evaluated whether the PB, OC, and GB extracts inhibited FFA-induced lipid accumulation in HepG2 cells using Nile red staining. Treatment with the PB, OC, and GB extracts dose-dependently reduced the intracellular lipid accumulation (Figure 1B). The presence of intracellular lipid droplets was also confirmed by Oil Red O staining. As expected, the treatment with the PB, OC, and GB extracts inhibited lipid droplet accumulation in the cytosol of HepG2 cells (Figure 1C). We further confirmed the PB, OC, and GB extract-mediated inhibition of FFA-induced lipid accumulation in HepG2 cells by quantification of intracellular TGs (Figure 1D). 

### 3.2. Effects of the PB, OC, and GB Extracts on Fatty Liver and Body Weight in the HFD-Induced NAFLD Mouse Model

**Figure 2:**
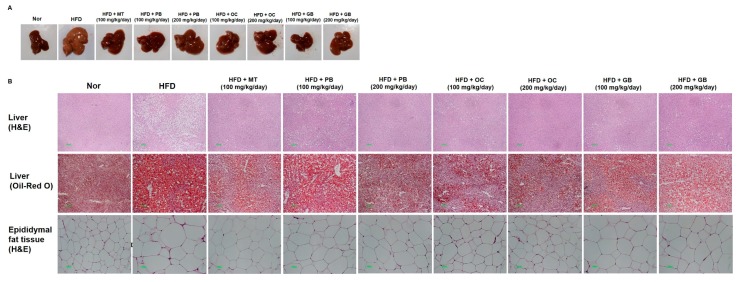

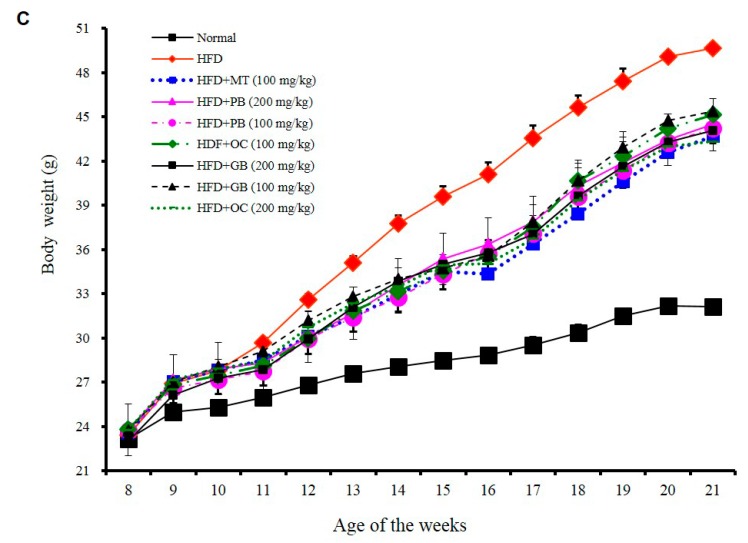

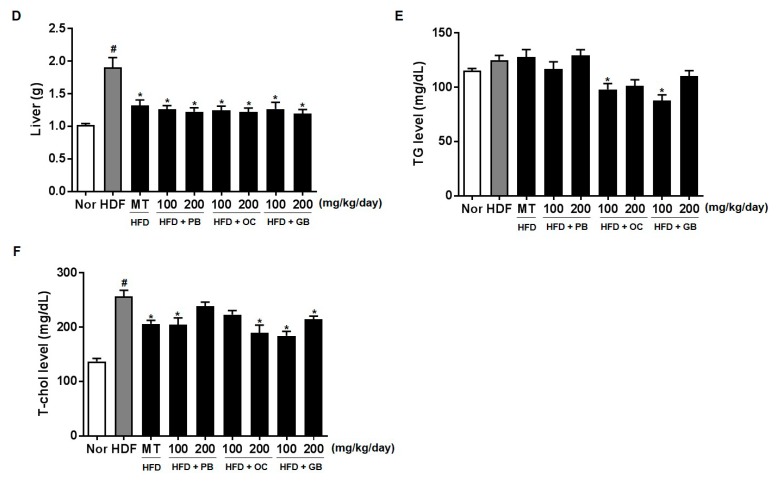
Effects of PB, OC, and GB extracts on mouse liver histology and body weight in a high-fat diet (HFD)-fed mouse model. (**A**) Photographs of the mouse liver. (**B**) Histopathological examination by hematoxylin and eosin (H&E) (liver and epididymis fat tissue) and Oil Red O (liver) staining and (**C**) body weights are shown for HFD-fed mice, with or without PB, OC, and GB extract supplementation. Liver weights (**D**), TG levels (**E**), and total cholesterol (TC) levels (**F**) in the liver tissues from the mice treated with the PB, OC, and GB extracts. MT, milk thistle. The data are presented as the mean ± SEM (*n* = 9); # *p* < 0.05 compared with the control group; * *p* < 0.05 compared with the HFD group.

To evaluate possible dose-related effects and protective mechanisms of the PB, OC, and GB extracts against HFD-induced fatty liver disease in vivo, we administered the PB, OC, and GB extracts to HFD-fed mice (100 mg/kg/day and 200 mg/kg/day), with one of the HFD group administered an MT extract (100 mg/kg/day) for a 14-week period. Consistent with previous reports, a 14-week HFD administration resulted in the accumulation of fat in the liver of the animals and in the development of liver steatosis (Figure 2A). On visual examination, the livers of the mice from the HFD group were pale yellow in appearance and markedly enlarged compared with those of the mice from the control group (Figure 2B). Liver histopathology revealed widespread lipid vacuolar deposits in the HFD group, with no such deposits in the control group. PB, OC, and GB extract treatments reduced this microvesicular fat accumulation. Oil Red O staining of liver sections confirmed a significantly higher accumulation of lipid droplets in the hepatocytes of the mice in the HFD group and its obvious decrease in the PB, OC, GB (100 mg/kg/day and 200 mg/kg/day), and MT extract-treated (100 mg/kg/day) groups. Also, the epididymal fat tissue size was larger in the HFD groups compared to the normal group, but it was smaller in the HFD groups treated with the PB, OC, GB (100 mg/kg/day and 200 mg/kg/day), and MT extracts (100 mg/kg/day).

Since HFD induced not only fatty liver but also obesity, we evaluated the chronic effects of the PB, OC, and GB extracts on the body weight. As shown in Figure 2C, the HFD-induced body weight gain was significantly reduced by daily oral administration of the PB, OC, GB (100 mg/kg/day and 200 mg/kg/day), or MT extracts (100 mg/kg/day), indicating that PB, OC, and GB extract treatments inhibited HFD-caused obesity. Particularly, the PB extract (200 mg/kg/day) was more effective than other extracts. However, the daily food and water consumptions were not significantly affected by PB, OC, and GB extract treatments.

In addition, PB, OC, and GB extract treatments attenuated the effects of HFD on the liver weight, with the weight restored to the normal control levels (Figure 2D). Therefore, PB, OC, and GB extract treatments significantly improved HFD-induced fatty liver and hepatomegaly. 

We further confirmed HFD-induced hepatic lipid accumulation by a quantitative analysis of TG and TC contents in the serum. Along with the hepatic steatosis phenotype, the serum TG and TC levels were substantially higher in the HFD group of mice than in the control group. A dose-related effect was confirmed, with a statistically significant decrease in the TG and TC levels in the mice receiving 100 mg/kg/day and 200 mg/kg/day of the PB, OC, and GB extracts (Figure 2E,F). 

**Figure 3:**
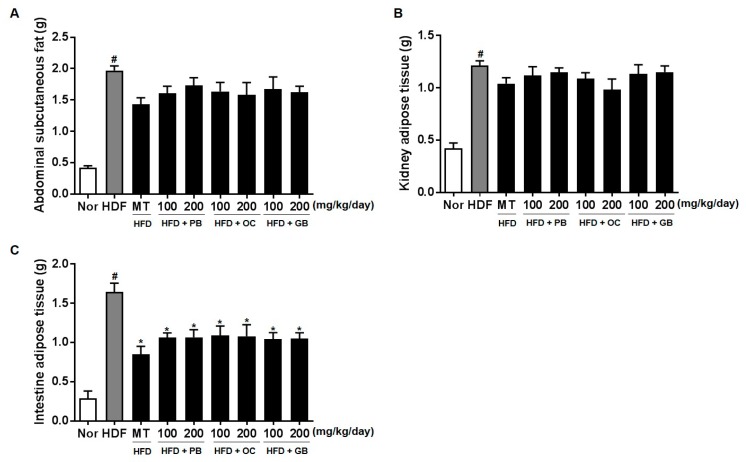
Effects of PB, OC, and GB extracts on body weight gain and body fat mass in HFD-fed mice: (**A**) abdominal subcutaneous fat, (**B**) kidney tissue, and (**C**) intestinal adipose tissue. The data are presented as the mean ± SEM (*n* = 9); # *p* < 0.05 compared with the control group; * *p* < 0.05 compared with the HFD group.

The weights of abdominal subcutaneous fat were higher in the HFD groups than in the control group (Figure 3A). Also, the weights of kidney adipose tissue and intestine adipose tissue were significantly decreased in the mice treated with the PB, OC, GB, or MT extract (Figure 3). 

### 3.3. Effects of the PB, OC, and GB Extracts on the Serum Levels of Liver Marker Enzymes

**Figure 4:**
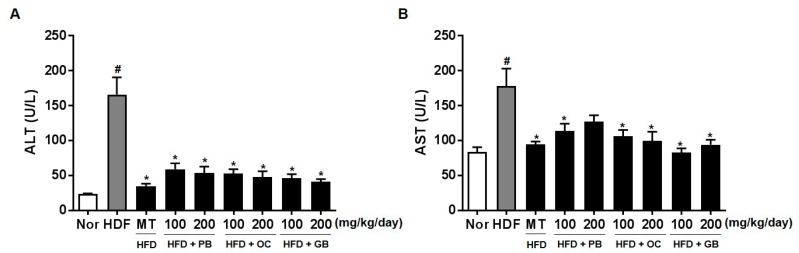
Effects of PB, OC, and GB extracts on serum alanine aminotransferase (ALT) and aspartate aminotransferase (AST) levels in HFD-fed mice. The serum ALT (**A**) and AST (**B**) levels were determined 14 weeks after PB, OC, and GB extracts treatment. The data are presented as the mean ± SEM (*n* = 9); # *p* < 0.05 compared with the control group; * *p* < 0.05 compared with the HFD group.

To evaluate the effect of the PB, OC, and GB extracts on liver function and damage, the serum enzyme activities of ALT and AST were measured. The serum ALT and AST levels were significantly higher in the mice from the HFD groups than in those from the control group (Figure 4). The HFD-induced increases in serum ALT and AST levels were dramatically suppressed by PB, OC, and GB extract treatments. 

**Figure 5:**
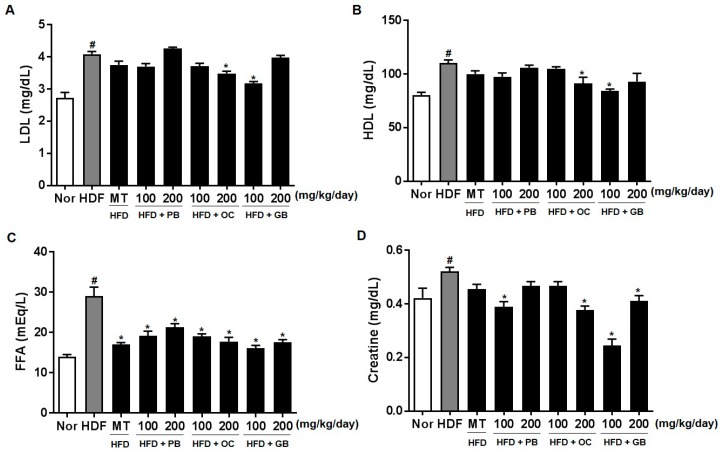

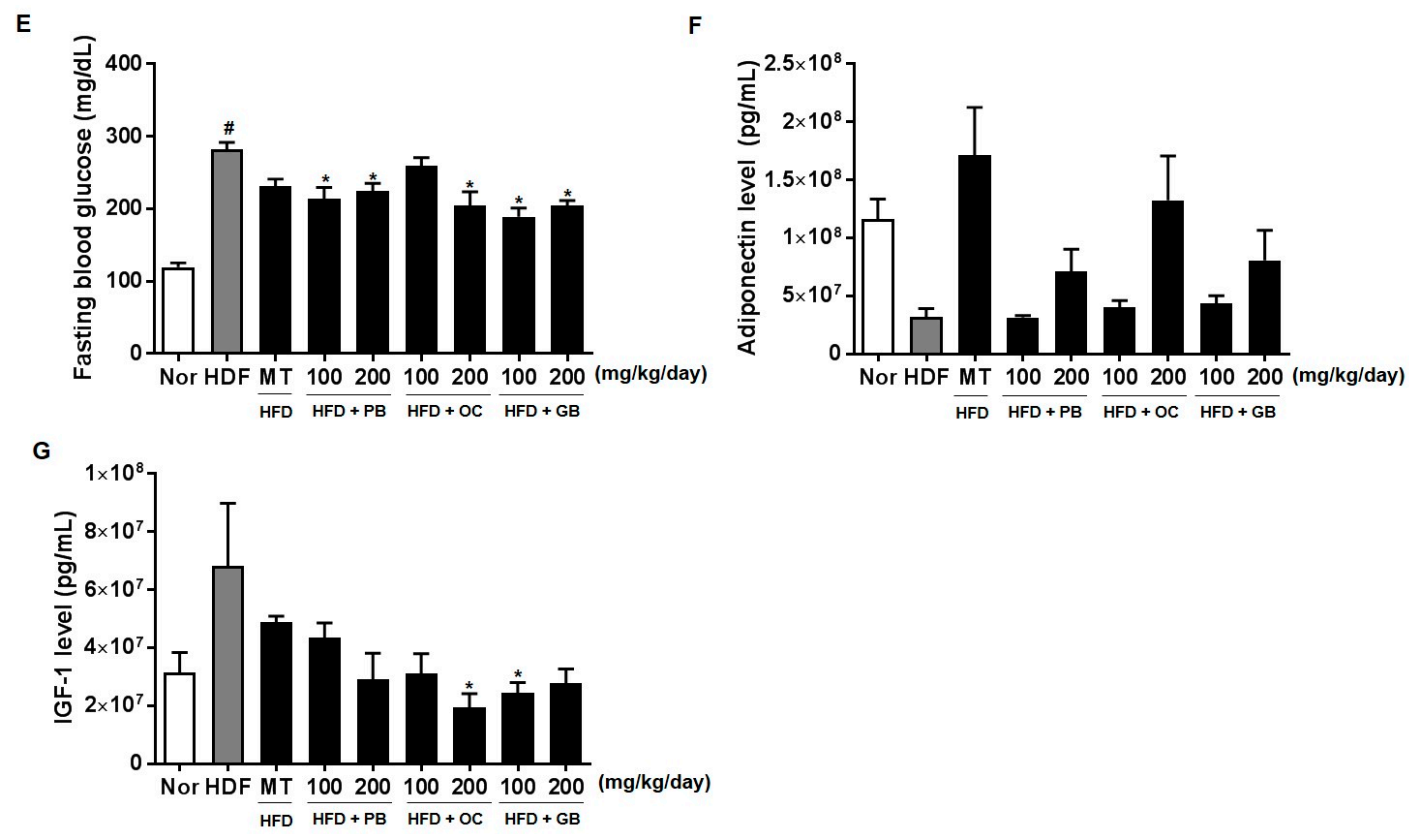
Effects of PB, OC, and GB extracts on blood biochemistry parameters in HFD-fed mice. (**A**) Serum low-density lipoprotein (LDL) cholesterol, (**B**) serum high-density lipoprotein (HDL) cholesterol, (**C**) serum FFAs, (**D**) serum creatine, (**E**) fasting blood glucose, (**F**) serum adiponectin, and (**G**) serum IGF-1 levels. The data are presented as the mean ± SEM (*n* = 9); # *p* < 0.05 compared with the control group; * *p* < 0.05 compared with the HFD group.

The effects of the PB, OC, and GB extracts on other serum parameters, such as LDL cholesterol, HDL cholesterol, FFAs, creatinine, fasting blood glucose, adiponectin, and IGF-1, were also investigated. As shown in Figure 5A–G, the increased levels of LDL cholesterol, FFAs, and blood glucose in the HFD mice were all significantly attenuated by PB, OC, and GB extract treatments. Although the serum creatinine and fasting blood glucose levels were increased by HFD, there were significant differences in their concentrations between the PB, OC, GB, and MT extract (100 mg/kg/day) groups and the HFD group (Figure 5D,E). In addition, the reduced serum adiponectin levels were markedly elevated by the PB, OC, and GB extracts (Figure 5F), whereas the HFD-related elevation of the serum levels of IGF-1 was suppressed by the PB, OC, and GB extracts in the HFD mice (Figure 5G). 

### 3.4. Effects of the PB, OC, and GB Extracts on the Expression of Genes Involved in Lipogenesis and Fatty Acid Uptake in the Liver

**Figure 6:**
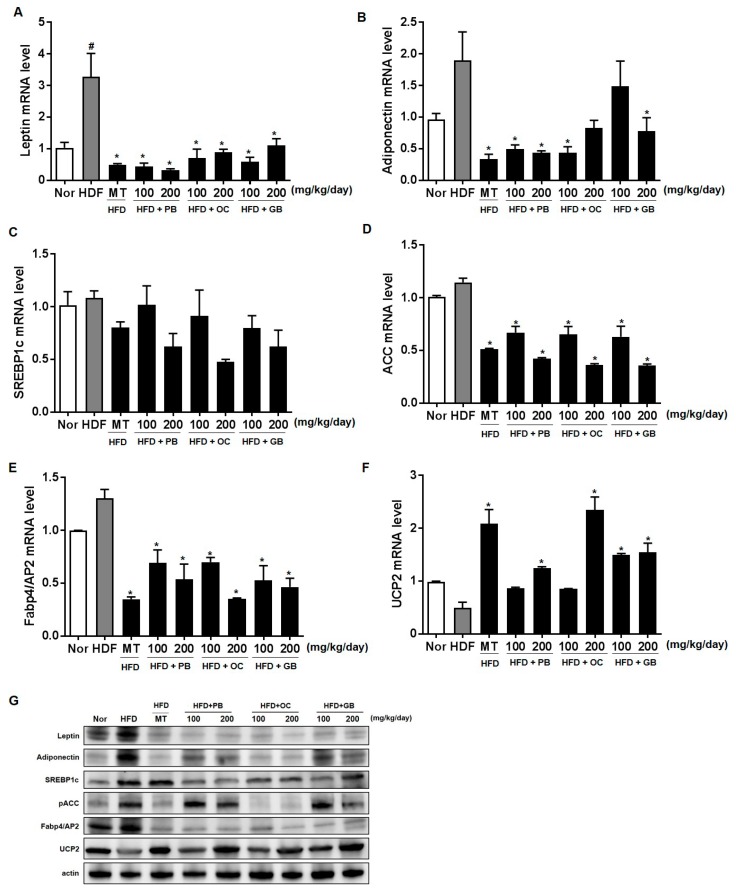
Effects of PB, OC, and GB extracts on the mRNA expression of lipogenesis-related genes in the liver of HFD-fed mice, determined by RT-PCR. Expression of (**A**) leptin, (**B**) adiponectin, (**C**) sterol regulatory element-binding protein-1 (SREBP-1), (**D**) acetyl-CoA carboxylase (ACC), (**E**) adipocyte protein 2 (AP2), and (**F**) mitochondrial uncoupling protein (UCP2) in the liver. (**G**) Western blot analysis of the corresponding proteins in HFD-fed mice livers. The data are presented as the mean ± SEM (*n* = 9); # *p* < 0.05 compared with the control group; * *p* < 0.05 compared with the HFD group.

To further confirm the protective effects of the PB, OC, and GB extracts against the HFD-induced hepatic steatosis, we performed real-time qPCR analysis of the expression of genes specific for lipid-associated pathways, using liver tissues from the mice that received HFD, with or without PB, OC, and GB extract supplementation. The levels of leptin expression significantly increased in the HFD group, with a notable comparative decrease in the liver of the mice treated with the PB, OC, and GB extracts (Figure 6A). On the contrary, adiponectin expression levels decreased in the HFD group and increased in the HDF mice treated with the PB, OC, and GB extracts (Figure 6B). Although the mRNA expression of sterol regulatory element-binding protein-1 (SREBP-1), acetyl-CoA carboxylase (ACC), and adipocyte protein 2 (AP2) were increased by HFD, supplementation with the PB, OC, GB, and MT extracts significantly reduced these mRNA levels in the liver (Figure 6C–E). Also, mitochondrial uncoupling protein (UCP2) was decreased by HFD, and supplementation with the PB, OC, GB, and MT extracts significantly increased its mRNA levels in the liver (Figure 6F). Western blot analysis confirmed that the protein expression of leptin, adiponectin, SREBP1c, ACC, and AP2 was higher in the HFD group and lower in the HDF mice treated with the PB, OC, and GB extracts. Also, HFD decreased UCP2 protein levels, and PB, OC, and GB increased UCP2 protein in the liver (Figure 6G). These results demonstrate that a reduction of lipogenesis was involved in the antilipidogenic effects of the PB, OC, and GB extracts. 

### 3.5. Effects of the PB, OC, and GB Extracts on the Expression of Genes Involved in Lipid Droplet Accumulation and Fatty Acid Uptake in the Liver

**Figure 7:**
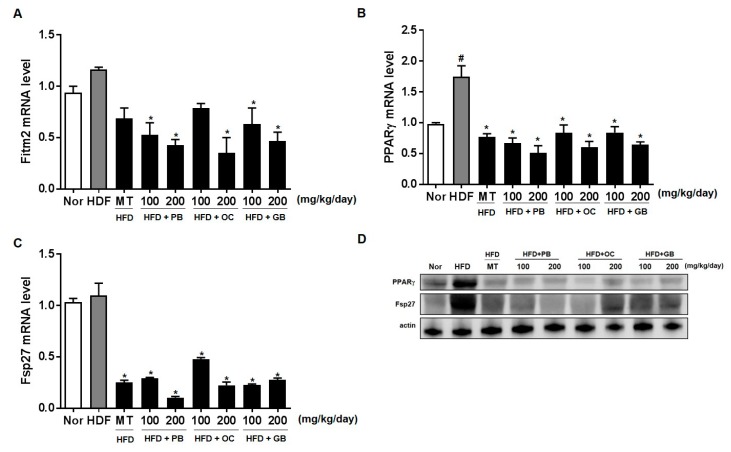
Effects of PB, OC, and GB extracts on the expression of lipogenesis genes in HFD-fed mice livers, determined by RT-PCR. (**A**) Fit2/Fitm2, (**B**) PPARγ, and (**C**) Fsp27/Cidec expression in the livers. (**D**) Western blot analysis of the corresponding proteins in HFD-fed mice livers. The data are presented as the mean ± SEM (*n* = 9); # *p* < 0.05 compared with the control group; * *p* < 0.05 compared with the HFD group.

To further quantify the protective effects of the PB, OC, and GB extracts on HFD-induced hepatic steatosis, we performed a lipid-associated pathway-specific quantitative real-time PCR analysis, using liver tissues from mice receiving a HFD, with or without PB, OC, and GB extracts supplementation. The levels of Fit2/Fitm2 and PPARγ were significantly increased in the HFD group, with a notable comparative decrease in the liver of mice treated with PB, OC, and GB extracts supplementation (Figure 7A,B). Fat-specific protein 27 (Fsp27, also known as Cidec), involved in lipid droplet accumulation, was increased in the HFD group and reduced in the HDF mice treated with PB, OC, and GB extracts (Figure 7C). Western blot analysis confirmed that protein (PPARγ and Fsp27) expression was higher in the HFD group and lower in the HDF group treated with the PB, OC, and GB extracts. (Figure 7D).

### 3.6. Effects of the PB, OC, and GB Extracts on the Expression of Genes Involved in Inflammation in the Liver and Adipose Tissue

**Figure 8:**
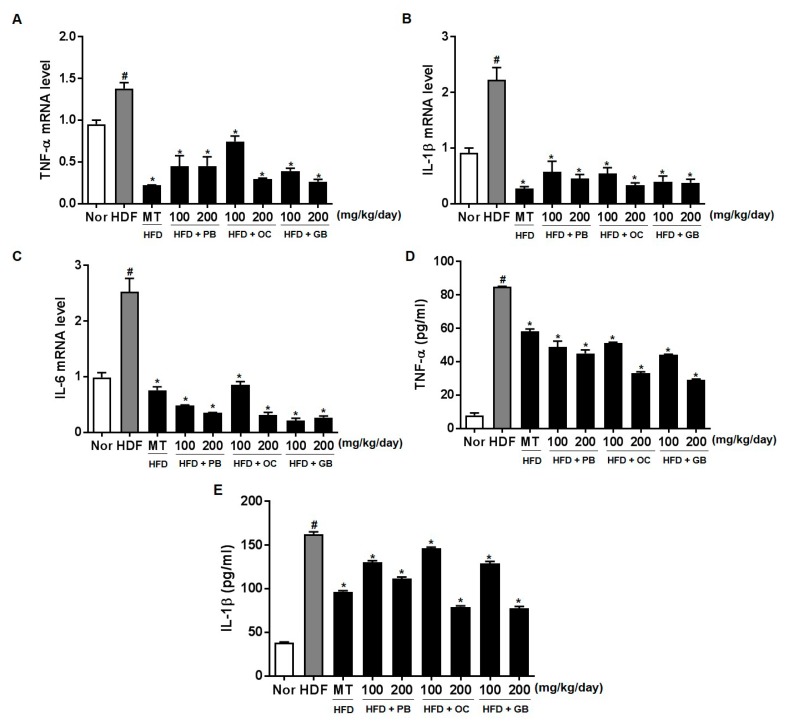
Effects of PB, OC, and GB extracts on the mRNA expression of inflammation-related genes in HFD-fed mice, determined by RT-PCR. (**A**) TNF-α, (**B**) IL-1β, and (**C**) IL-6 expression in the liver. Expression of (**D**) TNF-α and (**E**) IL-1β in the adipose tissue determined by ELISA assay. The data are presented as the mean ± SEM (*n* = 9); # *p* < 0.05 compared with the control group; * *p* < 0.05 compared with the HFD group.

To further quantify the protective effects of PB, OC, and GB extracts on HFD-induced inflammation, we performed a quantitative real-time PCR analysis using liver tissues and an ELISA assay for adipose tissues from mice receiving a HFD, with or without PB, OC, and GB extracts supplementation. The mRNA levels of TNF-α, IL-1β, and IL-6 were increased in the HFD group, with a notable comparative decrease in the liver of mice receiving PB, OC, and GB extracts supplementation (Figure 8A–C). Also, TNF-α and IL-1β were increased in the HFD group, with a notable comparative decrease in the adipose tissue of mice treated with PB, OC, and GB extracts supplementation (Figure 8D,E).

## 4. Discussion

Insects have attracted considerable interest in the fields of nutrition and toxicology because of the global exhaustion of food as a result of the growing population [15]. In previous studies, many insects, which are traditional, readily available, and nutritious foods, have shown no systemic toxicological effects [16,17]. Recent studies have confirmed that insects can be used as medicinal foods and health supplements [18]. The aim of our study was to evaluate the hepatoprotective effects of ethanol extracts of three insects, namely, *P. brevitarsis seulensis*, *O. chinensis sinuosa*, and *G. bimaculatus*, in an HFD-induced animal NAFLD model.

NAFLD is an acquired metabolic stress-related liver disorder, which was originally considered to largely occur in residents of wealthy industrialized Western countries [19]. The pathogenesis of NAFLD is often described in terms of the “first hit” and the “second hit”, with the “first hit” being lipid accumulation in the liver, and the “second hit” being characterized by proinflammatory mediators-induced inflammation, hepatocellular injury, and fibrosis [20]. NAFLD cases may progress through stages of simple, bland steatosis, nonalcoholic steatohepatitis (NASH), hepatic fibrosis, cirrhosis, and, finally, hepatocellular carcinoma [21]. 

To develop a novel, non-cytotoxic herbal medicine to treat NAFLD, PB, OC, and GB extracts were screened as potential lipid-lowering agents. In this study, we used an in vitro cellular model of hepatic steatosis to investigate whether PB, OC, and GB extracts have hepatoprotective activity in cells with FFA overload-induced steatosis. Furthermore, we examined changes in the cellular lipid content using Oil Red O staining and by measuring intracellular TG levels. The data showed that the PB, OC, and GB extracts reduced lipid accumulation in hepatocytes by regulating genes important for hepatic lipid droplet accumulation and FFA uptake. Lipid accumulation in the liver results from an imbalance between lipid deposition and removal, driven by hepatic synthesis of triglycerides, dietary intake of lipids, and de novo lipogenesis. The diet and relevant weight loss play an important role in the pathogenesis of nonalcoholic fatty liver disease (NAFLD) [22]. There is a report about the association between weight loss and changes in histological features of NASH in a prospective study of 293 patients [23], and there is growing evidence that weight loss would be a good strategy for the management of NAFLD [23,24]. Our results showed that a nutritional intervention with insect extracts reduced body weight, body fat mass, and lipid accumulation in the liver, improving the liver function.

TG accumulation in the cytoplasm of hepatocytes, a hallmark of NAFLD, arises from an imbalance between lipid uptake and removal and accompanies multiple pathophysiological processes in NASH [25]. Hepatic TG levels, TC, and liver weight were increased by HFD but attenuated by PB, OC, and GB extract treatments. Therefore, the PB, OC, and GB extracts exert critical effects, protecting against long-term HFD-induced liver damage. Although weight gain increased in all mice fed HFD, it was lower in the mice treated with the PB, OC, and GB extracts than in those from the HFD control group. Additionally, the weights of the body fat, abdominal subcutaneous fat, epidermal adipose tissue, and intestinal adipose tissue were reduced in the HFD-fed mice treated with the PB, OC, and GB extracts.

Liver biopsies from patients with persistently elevated liver enzyme levels indicated that elevated serum ALT and AST levels are useful in diagnosing NAFLD because they are primary laboratory abnormalities in chronic liver diseases [26,27]. We also observed high plasma ALT and AST levels in the HFD group and their significant decreases in the PB, OC, and GB extract-treated groups. The effects of the PB, OC, and GB extracts were also investigated on serum lipid parameters, including LDL and HDL cholesterol. As shown in Figure 5, increased levels of LDL and HDL cholesterol were attenuated by PB, OC, and GB extract treatments in HFD-fed mice. Excess intraabdominal fat, in particular, may be a key determinant in the pathogenesis of NAFLD because of its strong association with insulin resistance and also because it is a possible source of FFAs [28]. Increased adipose energy storage in obesity results in an increased FFA flux to other tissues and increased TG storage in these tissues, which promotes insulin resistance and other adverse effects [29]. The effects of the PB, OC, and GB extracts were also investigated on other serum parameters, such as LDH cholesterol, creatine, fasting blood glucose, non-essential fatty acids, adiponectin, IGF-1, and leptin. In this study, the blood glucose and IGF-1 levels were attenuated by PB, OC, and GB extract treatments in the HFD-fed mice, whereas the reduced serum adiponectin levels were markedly elevated by the PB, OC, and GB extracts. 

As adipocytes expand with TG accumulation, leptin secretion proportionally increases, and leptin stimulates fatty acid oxidation. Thus, adipocytes oxidize rather than store fat if the endogenous leptin they synthesize acts on the cells [30]. Therefore, we examined the effects of PB, OC, and GB extract supplementation on adipokine production, including leptin and adiponectin. It was demonstrated that HFD feeding caused a significant decrease in serum adiponectin levels, whereas leptin levels increased. However, PB, OC, and GB extract supplementation substantially attenuated both abnormalities. 

NAFLD is also intimately associated with a progressive manifestation of insulin resistance in the liver [31]. Increased glucose and insulin levels stimulate de novo lipogenesis via hepatic transcription factors such as SREBP-1. Activated by downstream insulin signaling, coupled with the X nuclear receptors in the liver, SREBP-1 decreases the expression of lipogenic enzymes, in particular ACC-1 [32]. Similar to adiponectin, leptin increases hepatic fatty acid oxidation and decreases de novo lipogenesis through phosphorylation of ACC-1 [33]. In NAFLD, fatty acids accumulate in hepatocytes because their de novo synthesis and uptake are upregulated in association with an increased expression of SREBP-1 and ACC-1 [34]. Lipid associated pathways such as leptin-, adiponectin-, SREBP-1-, ACC-, AP2-, and PPARγ-dependent pathways, were restored by the extracts, and the lipid accumulation of TG and TC in the serum decreased, resulting in liver and adipose weight loss. Adipose tissue can release free fatty acids (FFA) to the liver, where the mRNA levels of leptin, adiponectin, SRPEP-1, ACC, and UCP2 are altered, decreasing LDL and cholesterol in the blood and anti-inflammatory processes related to IL-1β, IL-6, and TNF-α [35]. Our results are consistent with the fact that the inflamed adipose tissue may drive FFAs specifically into the liver and not to other sites, determining, therefore, a specific phenotype at higher cardiovascular risk [36]. The therapeutic function of the examined insect extracts seems to be related to the regulation of insulin-, inflammation-, and lipogenesis associated pathways.

To gain further insights into preventive effects of the PB, OC, and GB extracts against HFD-induced hepatic steatosis, we performed real-time qPCR analysis of the expression of genes specific for lipid-associated pathways, using liver tissues from HFD-fed mice treated with the PB, OC, and GB extracts. The antilipidogenic effects of the PB, OC, and GB extracts were confirmed by the reduced expression of lipogenesis-related genes. Meanwhile, the expression of a fatty acid oxidation-related gene encoding UCP2 was increased by PB, OC, and GB extract supplementation. 

We also examined the levels of fat-inducing transcript 2 (Fit2), also known as fat storage-inducing transmembrane protein 2 (Fitm2), PPARγ, involved in the microvesicular accumulation of lipid droplets, and Fsp27/Cidec, an important regulator of energy homeostasis, related to the development of metabolic disorders, including obesity, diabetes, and liver steatosis [37]. The levels of Fit2/Fitm2, PPARγ, and Fsp27/Cidec were significantly increased in the HFD group, with a notable comparative decrease in the liver of mice treated with PB, OC, and GB extracts supplementation.

Also, adipose tissue inflammation leads to increased efflux of FFA that are subsequently taken up by the hepatocytes and drive triglyceride synthesis and accumulation [36]. There is a relationship between liver fat and adipose tissue inflammation, and inflammatory cytokines in the adipose tissue correlate with pathological changes in the liver in NAFLD [38]. In this study, the mRNA levels of TNF-α, IL-1β, and IL-6 increased in the HFD group, with a notable comparative decrease in the liver and adipose tissue of mice treated with PB, OC, and GB extracts supplementation.

## 5. Conclusions

In conclusion, we showed that PB, OC, and GB extracts ameliorated the body weight gain without inhibiting the appetite of HFD-fed mice. A daily oral supplementation with PB, OC, and GB extracts attenuated hepatic fat accumulation and lowered the blood levels of TGs, cholesterol, and FFAs via repression of genes involved in lipid import and lipid droplet accumulation. The sustained anti-NAFLD effects of the PB, OC, and GB extracts included alleviation of hepatic lipid accumulation, improvement of obesity and hepatic hypertriglyceridemia, and reduction of FFA uptake. Taken together, our results provide information on the pharmacological function of novel decoctions and support the potential usefulness of PB, OC, and GB extracts as effective novel anti-NAFLD drugs.
